# Mercapturic Acids Derived from the Toxicants Acrolein and Crotonaldehyde in the Urine of Cigarette Smokers from Five Ethnic Groups with Differing Risks for Lung Cancer

**DOI:** 10.1371/journal.pone.0124841

**Published:** 2015-06-08

**Authors:** Sungshim L. Park, Steven G. Carmella, Menglan Chen, Yesha Patel, Daniel O. Stram, Christopher A. Haiman, Loic Le Marchand, Stephen S. Hecht

**Affiliations:** 1 Department of Preventive Medicine, Norris Comprehensive Cancer Center, Keck School of Medicine, University of Southern California, Los Angeles, California, United States of America; 2 Masonic Cancer Center, University of Minnesota, Minneapolis, Minnesota, United States of America; 3 University of Hawaii Cancer Center, Honolulu, Hawaii, United States of America; New York University School of Medicine, UNITED STATES

## Abstract

The Multiethnic Cohort epidemiology study has clearly demonstrated that, compared to Whites and for the same number of cigarettes smoked, African Americans and Native Hawaiians have a higher risk for lung cancer whereas Latinos and Japanese Americans have a lower risk. Acrolein and crotonaldehyde are two important constituents of cigarette smoke which have well documented toxic effects and could play a role in lung cancer etiology. Their urinary metabolites 3-hydroxypropylmercapturic acid (3-HPMA) and 3-hydroxy-1-methylpropylmercapturic acid (HMPMA), respectively, are validated biomarkers of acrolein and crotonaldehyde exposure. We quantified levels of 3-HPMA and HMPMA in the urine of more than 2200 smokers from these five ethnic groups, and also carried out a genome wide association study using blood samples from these subjects. After adjusting for age, sex, creatinine, and total nicotine equivalents, geometric mean levels of 3-HPMA and HMPMA were significantly different in the five groups (P<0.0001). Native Hawaiians had the highest and Latinos the lowest geometric mean levels of both 3-HPMA and HMPMA. Levels of 3-HPMA and HMPMA were 3787 and 2759 pmol/ml urine, respectively, in Native Hawaiians and 1720 and 2210 pmol/ml urine in Latinos. These results suggest that acrolein and crotonaldehyde may be involved in lung cancer etiology, and that their divergent levels may partially explain the differing risks of Native Hawaiian and Latino smokers. No strong signals were associated with 3-HPMA in the genome wide association study, suggesting that formation of the glutathione conjugate of acrolein is mainly non-enzymatic, while the top significant association with HMPMA was located on chromosome 12 near the *TBX3* gene, but its relationship to HMPMA excretion is not clear.

## Introduction

Lung cancer is the leading cause of cancer death in the world, responsible for 1,590,000 deaths in 2012, about 4400 per day [1]. Cigarette smoking is the cause of approximately 80% of this mind-boggling death toll in males and at least 50% in females [2]. Decreasing the prevalence of cigarette smoking is one proven approach to lung cancer prevention; a goal would be to return lung cancer to the category of a relatively rare disease, as it was early in the 20^th^ century [3]. But the world has 1.25 billion smokers [4], whose nicotine addiction is eagerly fed and supported by tobacco companies with massive financial resources, so it does not appear that this goal will be reached in the near future. In the meantime, it is important to understand factors that dictate susceptibility to lung cancer, so that alternative preventive measures can be devised.

One clue to a better understanding of lung cancer susceptibility is different risks among smokers in varied ethnic groups. Thus, investigators in the Multi-ethnic Cohort (MEC) Study found that, for the same quantity of cigarettes smoked, African Americans and Native Hawaiians were at greater risk for lung cancer than Whites while Latinos and Japanese Americans were less susceptible [5]. These differences were evident in men and women and for all histologic types of lung cancer. The differences in susceptibility were most pronounced at lower numbers of cigarettes smoked per day, and were not observed in non-smokers. Many studies comparing lung cancer risk between specific ethnic groups have produced similar results [6–15].

**Fig 1:**
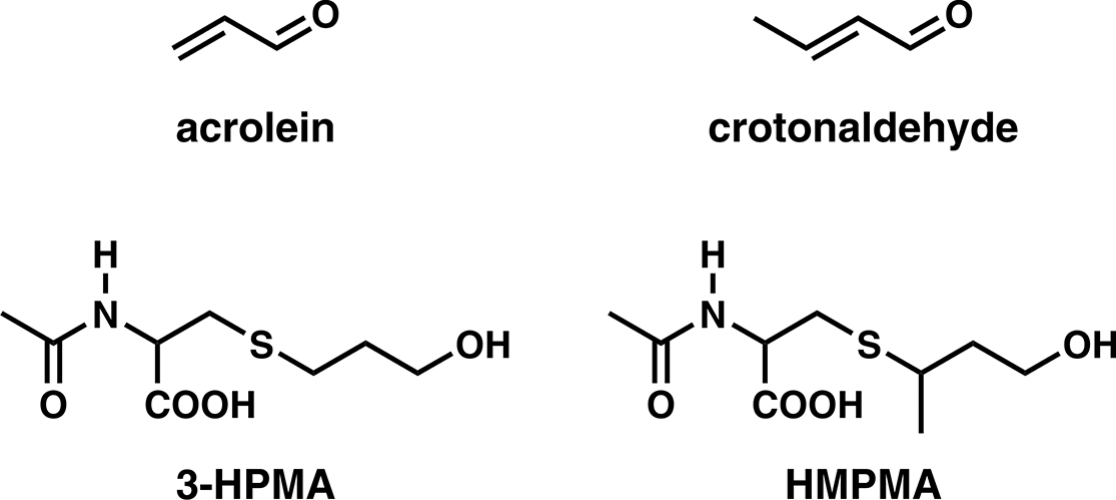
Structures of acrolein, crotonaldehyde, 3-HPMA, and HMPMA.

Our working hypothesis is that differences in the uptake and metabolism of pulmonary carcinogens and toxicants in tobacco smoke are responsible, at least in part, for the observed variation in lung cancer risk. We are exploring this hypothesis by analyzing tobacco smoke constituents and their metabolites in the urine of subjects from the five ethnic groups noted above in tandem with a genome wide association study (GWAS). In studies completed so far, we have reported differences in levels of nicotine and its metabolites as well as metabolites of the tobacco-specific lung carcinogen 4-(methylnitrosamino)-1-(3-pyridyl)-1-butanone (NNK) in these five ethnic groups and have examined the relationship of nicotine metabolites to GWAS signals on chromosome 4, specifically variants in *UGT2B10* [16–18]. In the study presented here, we have explored the possible roles of acrolein and crotonaldehyde by analysis of their metabolites 3-hydroxypropyl mercapturic acid (3-HPMA) and 3-hydroxy-1-methylpropylmercapturic acid (HMPMA), respectively, in urine [19,20]. Structures of these compounds are shown in **Fig 1**. 3-HPMA and HMPMA are formed by conjugation of acrolein and crotonaldehyde respectively with cellular glutathione, followed by metabolism of the glutathione conjugates and excretion in urine.

Both acrolein and crotonaldehyde are intense eye and respiratory tract irritants [21,22]. Consistently, inhalation studies of acrolein in laboratory animals demonstrate a variety of toxic effects including irritation, inflammation, cell proliferation, squamous metaplasia and other effects [21]. The irritant properties of crotonaldehyde are also well established [22]. Both acrolein and crotonaldehyde react with DNA to form cyclic 1,*N* ^*2*^-deoxyguanosine adducts and related structures [23,24]. The cyclic adducts have been detected in lung tissue from smokers [25,26], and the DNA binding pattern of acrolein in the *p53* tumor suppressor gene is similar to the pattern of mutational hotspots in *p53* found in lung tumors from smokers [27]. There is debate about the role of acrolein in lung carcinogenesis by cigarette smoke, as there is little evidence for its carcinogenicity in laboratory animals [21,28]. The irritant and inflammatory properties of both of these α,β-unsaturated aldehydes, along with the *p53* data for acrolein, indicate that they may play a role in lung carcinogenesis in smokers. In view of these facts, we have analyzed HPMA and HMPMA in the urine of self-identified African Americans, Native Hawaiians, Whites, Latinos, and Japanese Americans, all of whom were regular cigarette smokers. A GWAS in search of common genetic variants possibly predictive of 3-HPMA and HMPMA levels in these subjects was also conducted.

## Materials and Methods

### Subjects

Approval for this study, including the consent procedure, was obtained from the Institutional Review Boards of the University of Minnesota, the University of Hawaii, and the University of Southern California. Study participants provided written consent. IRB Code Number: 0912M75654. Subjects took part in the MEC, a prospective cohort study investigating the association of genetic and lifestyle factors with chronic diseases in a population with diverse ethnic backgrounds [29]. The cohort consists of 215,251 men and women, ages 45 to 75 at baseline, belonging mainly to the following five ethnic/racial groups: African Americans, Native Hawaiians, Whites, Latinos, and Japanese Americans. Potential participants were identified between 1993 and 1996 in Hawaii and California (mainly Los Angeles County) through voter registration lists, drivers’ license files, and Health Care Financing Administration data. Each participant completed a self-administered questionnaire which was delivered by mail and inquired about demographic, dietary, lifestyle, and other exposure factors.

This specific study was carried out in a subgroup of the MEC participants who were current smokers and were cancer-free at the time of urine collection. Thus, about 10 years after their entry into the cohort, 2,393 of this subgroup participated in the MEC bio-specimen sub-cohort and provided a blood sample and a first morning urine sample (subjects recruited in California) or overnight urine sample (subjects recruited in Hawaii), and completed an epidemiologic questionnaire, smoking history questionnaire and medication record. The overnight urine sample was collected starting between 5 pm and 9 pm (depending on the subject). This sample includes all urine passed during the night as well as the first morning urine. All urine was kept on ice until processing. Aliquots were subsequently stored in a -80°C freezer until analysis. The overnight or first morning urine was used to measure 3-HPMA and HMPMA.

### Analysis of 3-HPMA and HMPMA

This was performed using a 96-well high throughput LC-MS/MS method, as described previously [30]. Detection limits were 4.5 pmol/ml for 3-HPMA and 3.5 pmol/ml for HMPMA. Accuracy was 92% for 3-HPMA and 97% for HMPMA. Inter-day precision was 9.1% [coefficient of variation (CV)] for 3-HPMA and 11.0% for HMPMA. Among blind duplicates included among the samples, there were inter-batch CVs of 18.9% for 3-HPMA and 19.6% for HMPMA while the intra-batch CVs were 9.2% and 7.7% for the respective metabolites.

### Total nicotine equivalents

Total nicotine equivalents, the sum of nicotine and its metabolites nicotine glucuronide, cotinine, cotinine glucuronide, 3′-hydroxycotinine, 3′-hydroxycotinine glucuronide, and nicotine *N*-oxide, were determined as described previously [16]. These data have been published [16], and were used here for statistical adjustments of the 3-HPMA and HMPMA data.

### Creatinine Analysis

Creatinine was analyzed using a colorimetric microplate assay (CRE34-K01) purchased from Eagle Bioscience (http://stores.eaglebio.com/creatinine-microplate-assay-kit).

### Statistical methods

For this analysis, 2,291 subjects were retained. These subjects had total nicotine equivalents >1.27 nmol/ml (4-times the limit of quantitation) [16] and had either 3-HPMA or HMPMA measured. Of the 2,291 subjects, nine subjects were missing measures of 3-HPMA and seven subjects were missing measures of HMPMA.

Additionally, among the subjects retained for this analysis, 11 participants were missing BMI and 42 participants had missing values for cigarettes per day (CPD) at the time of urine collection. Using the Markov Chain Monte Carlo method and PROC MI statement from the SAS v9.2 software (SAS institute, Cary, NC)[31], the missing values for BMI and CPD were imputed. The imputed values were based on age at cohort entry, race/ethnicity, time between cohort entry and time of urine collection, BMI at baseline or number of CPD at baseline and smoking duration (for missing CPD). Ten datasets were created for the imputed missing values and the mean values across all 10 datasets were used to replace the missing measures.

To examine the correlation between 3-HPMA, HMPMA and measures of smoking (CPD and total nicotine equivalents), Pearson’s partial correlation coefficients (r) were adjusted for age, sex and race/ethnicity and creatinine levels (natural log). To compare the rank of 3-HPMA and HMPMA levels across race/ethnicity, the Wilcoxon Mann-Whitney test was employed. Also, the covariate-adjusted geometric means for 3-HPMA and HMPMA were computed for each ethnic/racial group at the mean covariate vector. We used two multivariable linear models. The first adjusted for the following predictors: age at time of urine collection (continuous), sex, race, and creatinine levels (natural log) and the second additionally adjusted for total nicotine equivalents. We also examined whether other factors such as BMI were associated with 3-HPMA and HMPMA. To better meet model assumptions, 3-HPMA and HMPMA were transformed by taking the natural log. For ease of interpretation, the values presented in the tables were back-transformed as geometric means to their natural scale.

### GWAS methods

A total of 2,418 current smokers were genotyped using the Illumina Human1M-Duo BeadChip (1,199,187 SNPs), as previously described [17]. Imputation of the variants included in the 1000 Genomes Project (http://www.1000genomes.org/) was performed using SHAPEIT [32] and IMPUTE2 [33] using a cosmopolitan reference panel (all groups included). After imputation with IMPUTE2 we used SNPs with an IMPUTE2 info score of ≥0.30 and minor allele frequency (MAF) >1% in any MEC ethnic group in our association testing. For 3-HPMA and HMPMA, a total of 2,211 and 2,213 study participants, respectively, with complete genotype and phenotype data, and 11,892,802 SNPs/indels (1,131,426 genotyped and 10,761,376 imputed) were included in the analysis. In the genetic analyses we adjusted for principal components, race, sex, age, creatinine and total nicotine equivalents, and enforced criteria of 5x10^-8^ for genome wide significance.

## Results

Characteristics of the subjects in this study are summarized in **Table 1**. Median ages in the 5 ethnic groups ranged from 60–64 years, BMI from 24.4–26.9 kg/m^3^, creatinine from 54–89 mg/dL, cigarettes per day from 7.1–20, and total nicotine equivalents from 27.2–44.4 nmol/mL. Males comprised 30.6% of the African Americans, 36.5% of the Native Hawaiians, 43.3% of the Whites, 51.5% of the Latinos, and 57.1% of the Japanese Americans. In all groups, males smoked more cigarettes per day than females, and males had higher levels of total nicotine equivalents than females in all five ethnic groups.

**Table 1:** Main characteristics of study participants^a^ stratified by race/ethnicity.

	African Americans		Native Hawaiians		Whites		Latinos		Japanese Americans		
		Median (Interquartile)									p-values
**All**		n = 363		n = 329		n = 441		n = 454		n = 704	
Age (years)	64	(59–69)	60	(56–65)	62	(58–68)	65	(61–70)	62	(58–69)	<0.0001
BMI (kg/m^2^)	26.9	(23.4–30.7)	26.8	(24.2–30.8)	24.7	(22.0–28.0)	26.5	(24.1–29.8)	24.4	(21.9–27.0)	<0.0001
Creatinine (mg/dl)	89	(54–142)	60	(38–91)	54	(33–85)	77	(50–117)	55	(34.0–88.5)	<0.0001
Cigarettes per day	10	(5–15)	15	(8–20)	20	(10–20)	7.1	(4–12)	12	(9–20)	<0.0001
Total nicotine equivalents (nmol/ml)	44.4	(27.4–74.1)	31.3	(20.0–48.0)	36.3	(22.0–61.1)	32.5	(20.9–53.6)	27.2	(15.8–43.5)	<0.0001
**Males**		n = 111		n = 120		n = 191		n = 238		n = 402	
Age (years)	63	(58–66)	63	(58–68)	62	(59–67)	65	(62–71)	62	(58–68)	<0.0001
BMI (kg/m^2^)	26.4	(23.1–28.5)	26.8	(24.3–30.6)	25.8	(23.3–28.7)	25.8	(23.7–28.7)	24.8	(22.7–27.4)	<0.0001
Creatinine (mg/dl)	124	(81.5–167)	75	(50.5–124.4)	71	(47–108)	90.5	(58.1–135)	70	(43.1–105)	<0.0001
Cigarettes per day	10	(6.301–20)	18.5	(10–20)	20	(15–25)	10	(5–15)	15	(10–20)	<0.0001
Total nicotine equivalents (nmol/ml)	54.4	(32.0–92.8)	33.4	(22.2–52.0)	41.2	(24.6–78.4)	35.0	(21.9–59.1)	30.0	(17.9–47.6)	<0.0001
**Females**		n = 252		n = 209		n = 250		n = 216		n = 302	
Age (years)	64	(59–71)	59	(56–64)	62	(58–69)	64.5	(60–69)	62	(58–69)	<0.0001
BMI (kg/m^2^)	27.4	(23.5–31.5)	26.8	(24.0–31.1)	23.9	(21.0–28.0)	27.1	(24.2–30.6)	23.5	(20.6–26.6)	<0.0001
Creatinine (mg/dl)	79.5	(48.3–126.5)	54	(33–79)	47	(29–67)	64.5	(44.8–93.5)	43.738	(28.0–66.2)	<0.0001
Cigarettes per day	10	(5–15)	12	(8–20)	15	(8–20)	6	(4.0–10)	10	(7–15)	<0.0001
Total nicotine equivalents (nmol/ml)	41.4	(26.0–66.3)	30.1	(19.3–45.3)	32.1	(20.5–50.7)	31.7	(18.5–50.3)	22.3	(13.7–35.6)	<0.0001

^a^ Includes never married, separated, widowed, and divorced.

Correlations among cigarettes per day, total nicotine equivalents, and levels of 3-HPMA and HMPMA are summarized in **Table 2**. All correlations were significant (p<0.0001). The strongest correlations were between 3-HPMA and HMPMA, r = 0.83–0.86, while correlations between total nicotine equivalents and the mercapturic acid levels were somewhat weaker, r = 0.52–0.6. Similar correlation coefficients were obtained when analyzed by ethnic group or gender, in all cases significant (p<0.0001).

**Table 2:** Pearson's correlation between measures of smoking and 3-HPMA and HMPMA.^a^

All N = 2221				Males N = 1031				Females N = 1190			
	CPD	TNE	3-HPMA		CPD	TNE	3-HPMA		CPD	TNE	3-HPMA
TNE	0.5			TNE	0.49			TNE	0.51		
p-value	<0.0001			p-value	<0.0001			p-value	<0.0001		
3-HPMA	0.32	0.53		3-HPMA	0.34	0.52		3-HPMA	0.31	0.53	
p-value	<0.0001	<0.0001		p-value	<0.0001	<0.0001		p-value	<0.0001	<0.0001	
HMPMA	0.35	0.58	0.85	HMPMA	0.37	0.6	0.83	HMPMA	0.33	0.58	0.86
p-value	<0.0001	<0.0001	<0.0001	p-value	<0.0001	<0.0001	<0.0001	p-value	<0.0001	<0.0001	<0.0001

^a^ Abbreviations. CPD, cigarettes per day; TNE, total nicotine equivalents; 3-HPMA, 3-hydroxypropylmercapturic acid; HMPMA, 3-hydroxy-1-methylpropylmercapturic acid

Medians and interquartile ranges for levels of 3-HPMA and HMPMA, expressed as pmol/ml urine, are summarized in **Table 3**. For both 3-HPMA and HMPMA, levels of these metabolites were highest and not significantly different among African Americans, Native Hawaiians, and Whites, whereas Latinos and Japanese Americans had significantly lower levels. The data are stratified by sex in **S1 Table**. Within each sex, the same trend was observed when comparing the ethnic groups. The relatively high values of 3-HPMA and HMPMA in Native Hawaiians were not due to outliers because they remained after removing the bottom and top 1% of both the total nicotine equivalents and mercapturic acid values. We also observed that levels of both 3-HPMA and HMPMA were significantly higher in males than in females in all ethnic groups (**S1 Table**). When expressed per total nicotine equivalents, 3-HPMA levels were highest in Native Hawaiians and Japanese Americans, and HMPMA was highest in Native Hawaiians (**Table 3**). When expressed per total nicotine equivalents, 3-HPMA and HMPMA levels were significantly higher in males than females in all ethnic groups (p<0.01) except HMPMA in Latinos (p = 0.14)(**S1 Table**).

**Table 3:** Median and interquartile range for measures of 3-HPMA and HMPMA.

	N	Median	(Interquartile range)	p-value^a^ when compared to whites
**3-HPMA (pmol/ml)**				
**African Americans**	362	3648	(1699–7328)	0.96
**Native Hawaiians**	329	3736	(2022–6526)	0.78
**Whites**	438	3548	(1949–6757)	
**Latinos**	449	2528	(1263–4855)	<0.0001
**Japanese Americans**	704	2955	(1568–5353)	0.002
**p-value**			<0.0001	
**HMPMA (pmol/ml)**				
**African Americans**	361	2948	(1418–5194)	0.16
**Native Hawaiians**	329	2766	(1473–4493)	0.67
**Whites**	440	2535	(1423–4492)	
**Latinos**	452	1986	(1079–3602)	<0.0001
**Japanese Americans**	702	2134	(1037–3507)	<0.0001
**p-value**			<0.0001	
**3-HPMA/TNE** ^**b**^ **(10** ^**3**^ **)**				
**African Americans**	362	80.90	(48.35–125.3)	<0.0001
**Native Hawaiians**	329	111.7	(83.58–150.4)	0.008
**Whites**	438	101.6	(70.89–143.6)	
**Latinos**	449	83.36	(54.83–125.5)	<0.0001
**Japanese Americans**	704	111.5	(77.76–169.0)	0.008
**p-value**			<0.0001	
**HMPMA/TNE (10** ^**3**^ **)**				
**African Americans**	361	63.66	(41.35–92.47)	<0.0001
**Native Hawaiians**	329	82.35	(62.13–113.5)	0.009
**Whites**	440	75.69	(53.21–106.6)	
**Latinos**	452	64.12	(43.14–96.39)	0.0002
**Japanese Americans**	702	78.86	(54.80–109.2)	0.49
**p-value**			<0.0001	
**3-HPMA (pmol/mg creatinine)**				
**African Americans**	362	4123	(2341–6808)	<0.0001
**Native Hawaiians**	329	6007	(3947–9606)	0.19
**Whites**	438	6738	(3885–1057)	
**Latinos**	449	3480	(1864–5908)	<0.0001
**Japanese Americans**	704	5344	(3163–8596)	<0.0001
**p-value**			<0.0001	
**HMPMA (pmol/mg creatinine)**				
**African Americans**	361	3240	(1886–5285)	<0.0001
**Native Hawaiians**	329	4404	(2753–6780)	0.1
**Whites**	440	4757	(3101–7215)	
**Latinos**	452	2613	(1526–4566)	<0.0001
**Japanese Americans**	702	3591	(2183–5513)	<0.0001
**p-value**			<0.0001	

^a^ p-value calculated using Wilcox Mann-Whitney test.

^b^ TNE, total nicotine equivalents.

Medians and interquartile ranges for levels of 3-HPMA and HMPMA, expressed per mg creatinine, are summarized in **Table 3**. Levels of 3-HPMA and HMPMA were highest in Whites and Native Hawaiians, with significantly lower levels in African Americans, Latinos, and Japanese Americans. The lower levels in African Americans when expressed in this manner are due to the significantly higher levels of creatinine in this group (**Table 1**).

**Fig 2:**
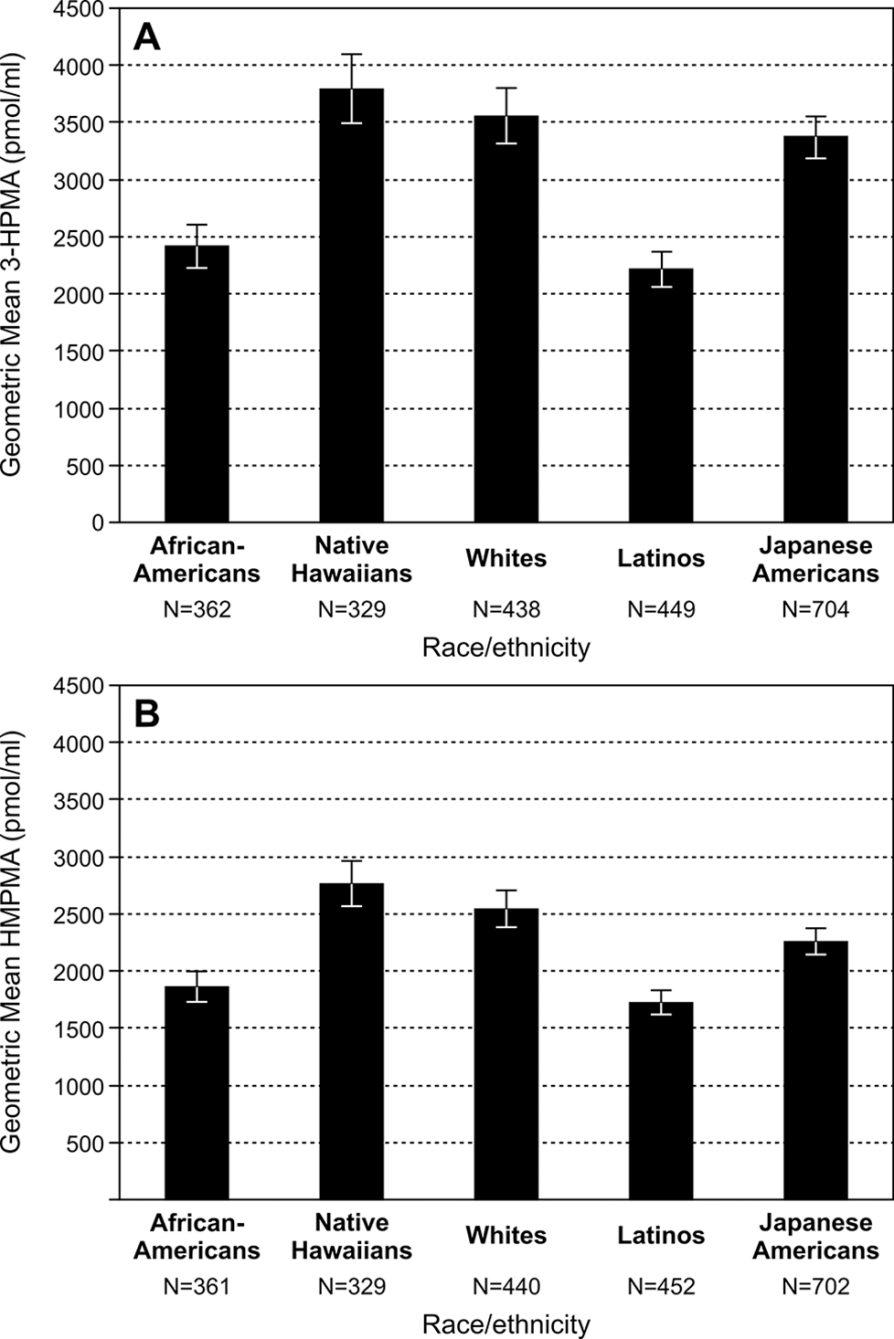
Geometric means of A. 3-HPMA and B. HMPMA in the five ethnic groups, after adjusting for age, sex, creatinine, and total nicotine equivalents.

Geometric means of 3-HPMA and HMPMA in the five ethnic groups are presented in **Table 4**. In Model 1, they have been adjusted for age, sex, and creatinine. For both 3-HPMA and HMPMA, the highest levels were in Whites and Native Hawaiians, with significantly lower levels in African Americans, Japanese Americans, and Latinos. The lowest levels of both mercapturic acids were in Latinos, and these were significantly lower than in all other groups. Model 2 was additionally adjusted for total nicotine equivalents; the results were similar to those in Model 1, except that the differences between the Latinos and African Americans were no longer significant for either mercapturic acid, and the difference in 3-HPMA levels in the Japanese Americans and Whites was no longer significant. These results are illustrated graphically in **Fig 2**.

**Table 4:** Geometric means (95% CIs) of 3-HPMA and HMPMA, stratified by race/ethnicity.

		Model 1						Model 2					
	N	Geometric means	(95% CI)					Geometric means	(95% CI)				
**3-HPMA (pmol/ml)**													
African Americans	362	2623	(2403–2864)†		§	*	y	2406	(2226–2600)†		§		y
Native Hawaiians	329	3689	(3373–4035)	‡		*	y	3787	(3499–4099)	‡		*	y
Whites	438	3985	(3690–4304)	‡		*	y	3549	(3314–3801)	‡		*	
Latinos	449	2087	(1933–2253)†	‡	§		y	2210	(2066–2365)†		§		y
Japanese Americans	704	3142	(2956–3340)†	‡	§	*		3369	(3191–3557)	‡	§	*	
p-value**			<0.0001						<0.0001				
**HMPMA (pmol/ml)**													
African Americans	361	2024	(1865–2196)†		§	*		1860	(1733–1997)†		§		y
Native Hawaiians	329	2689	(2474–2922)	‡		*	y	2759	(2567–2965)	‡		*	y
Whites	440	2856	(2659–3068)	‡		*	y	2541	(2388–2705)	‡		*	y
Latinos	452	1624	(1513–1743)†	‡	§		y	1720	(1618–1829)†		§		y
Japanese Americans	702	2108	(1992–2232)†		§	*		2259	(2150–2373)†	‡	§	*	
p-value**			<0.0001						<0.0001				

Model 1, adjusted for age, sex, creatinine

Model 2, additionally adjusted for total nicotine equivalents

† significant when compared to whites

‡ significant when compared to African Americans

§ significant when compared to Native Hawaiians

* significant when compared to Latinos

y significant when compared to Japanese Americans

** global p-value

In the GWAS analysis of 3-HPMA and HMPMA we observed little evidence of inflation in the test statistic in the overall multiethnic sample (λ = 1.0; **S1** and **S2 Figs**) or in any single ethnic group (0.95 ≤ λ’s ≤ 1.0) for either phenotype. There were no globally significant variants for the overall results for 3-HPMA using our genomic threshold of p <5x10^-8^ (**S2 Table**). The overall results for HMPMA showed a total of nine globally significant variants (p-values ranged from 4.3x10^-8^ to 9.7x10^-10^) on seven different chromosomes; all of these SNPs are common in African Americans, two are also common in Whites and two others in Latinos (**S3 Table**). The top significant association, rs55922880 (chr12), is located near gene *TBX3*, a gene involved in encoding transcription factors. Together, these nine variants explain only 5.4% of variability in HMPMA overall, but when observed by ethnic group, they explain 15.8% in African Americans, 11.7% in Latinos followed by 2.8% in Native Hawaiians and 2.1% in Whites with the least variability explained in the Japanese Americans at 1.4%.

In ethnic specific analyses for both phenotypes there were widely scattered associations that were often difficult to interpret due to very low minor allele frequencies (**S4**, **S5**, **S6**, **S7**, **S8**, **S9**, **S10**, **S11**, **S12**, **S13 Tables**). Of the nine overall significant variants for HMPMA, only one (rs7675915) was also found to be globally significant in the ethnic specific analysis (among Latinos at 2.95 x 10^–8^). However, all of the associations tended to be consistent by the different ethnic groups.

## Discussion

The urine samples in this study have been previously analyzed for total nicotine equivalents and total NNAL [16,18]. Total nicotine equivalents is an excellent indicator of nicotine dose in smokers [34]. Total NNAL (the sum of free NNAL and its glucuronides) correlates with total nicotine equivalents [18]. Free NNAL and NNAL glucuronides are metabolites of the potent tobacco-specific lung carcinogen NNK [35]. Free NNAL is itself a powerful lung carcinogen [36,37]. In these studies, median levels of total nicotine equivalents and total NNAL, expressed per ml urine, were highest in African Americans, intermediate in Whites, and lowest in Japanese Americans, and these differences were significant [16,18]. This order of total nicotine equivalents and total NNAL concentrations is consistent with the lung cancer risk for African Americans (highest), Whites (intermediate), and Japanese Americans (lowest) [5]. But the data for Native Hawaiians and Latinos in those two studies did not reflect their lung cancer risk. Native Hawaiians had significantly lower levels of total nicotine equivalents and total NNAL than Whites, when expressed as medians per ml urine, and Latinos had levels the same as Whites [16,18]. The results of the study reported here, in Model 2 (**Table 4 and Fig 2**), demonstrate that Native Hawaiians had the highest levels of the acrolein and crotonaldehyde metabolites 3-HPMA and HMPMA among the five ethnic groups, and these were statistically indistinguishable from those of Whites, and significantly higher than those of the other groups. Collectively, our results to date thus suggest that acrolein and crotonaldehyde may play some role in the relatively high lung cancer risk of Native Hawaiians. Furthermore, the relatively low levels of 3-HPMA and HMPMA in Latinos are also consistent with their lower lung cancer risk.

All humans have 3-HPMA and HMPMA in their urine because acrolein and crotonaldehyde are ubiquitous environmental and dietary constituents as well as being products of endogenous metabolism. Levels of 3-HPMA are typically about 4–10 times higher in the urine of smokers than non-smokers and they decrease rapidly and significantly when people stop smoking [19,38–43]. Similar findings pertain to HMPMA but fewer studies have been reported [19,40,41,44]. Cigarette mainstream smoke typically contains 5–60 μg of acrolein per cigarette, and these levels as well as those of nicotine correlate with “tar” in the same brands [45]; less data are available for crotonaldehyde. “Mouth level exposure” to acrolein in cigarette smokers, as determined in cigarettes with increasing deliveries of acrolein, is highly correlated with 3-HPMA in urine [46]. Similarly, mouth level exposure to nicotine is highly correlated with nicotine equivalents in urine [46]. Furthermore, in our study and in a large population based study, total nicotine equivalents in urine correlated with 3-HPMA in urine [47]. Collectively, these observations indicate that acrolein and nicotine in cigarette smoke as well as total nicotine equivalents in urine are strong determinants of 3-HPMA in urine. The Native Hawaiians in our study seem to represent an exception to this generality as their total nicotine equivalents were significantly lower than those of African Americans (p<0.0001) or Whites (p = 0.0145) [16], yet their 3-HPMA levels were as high as those of African Americans and Whites. This suggests that there is an important source of acrolein exposure in Native Hawaiians, either exogenous or endogenous, other than cigarette smoke, that contributes to their relatively high levels of urinary 3-HPMA and possibly to lung cancer risk. Similar considerations would presumably apply to HMPMA. We do not have data on the types of cigarettes smoked by the Native Hawaiians in this study, nor do we have data on 3-HPMA or HMPMA in Native Hawaiian non-smokers compared to non-smokers from other ethnic groups. Such data could possibly provide further insight into their 3-HPMA levels.

Previous studies have clearly established the presence of 3-HPMA in the urine of all non-smokers, but the levels can be variable [19]. In one study, a non-smoker group had levels of 3-HPMA which were more than 5 times higher than those reported in some large studies of smokers, indicating important sources of acrolein exposure other than tobacco smoke, but these were not identified [48]. Forest fires, urban fires, automobile exhaust, cooking fumes, and other sources of incomplete combustion including industrial emissions are among the environmental sources of acrolein [21,49–51]. It has also been detected in certain foods and beverages such as coffee and tea [21] and is produced in the body during lipid peroxidation, amino acid metabolism, and polyamine metabolism [52]. Sources of exposure to crotonaldehyde are similar to those of acrolein, and there is convincing evidence from studies of DNA adducts that crotonaldehyde is formed endogenously in humans [22,53,54].

The toxic effects of acrolein, an intensely irritating compound with a disagreeable acrid and pungent odor, are well documented [21,49]. Inhalation of acrolein causes severe respiratory distress and a wide variety of toxic effects in laboratory animals including toxicity to cilia, depressed respiratory rate, weight loss, inflammation, immunosuppression, cell proliferation, and various histopathological changes in the respiratory tract [21,49,52,55]. Acrolein reacts easily with critical proteins such as thioredoxin reductase in bronchial epithelial cells, resulting in dysregulation of cellular oxidative balance and related effects [56–58]. It also inhibits acetylation of aromatic amines and nucleotide excision repair [59,60]. Carcinogenicity studies of acrolein alone have been uniformly negative [21]. One study demonstrated a significantly increased incidence of bladder tumors in rats treated by i.p. injection with acrolein followed by dietary uracil [21]. In spite of these relatively negative carcinogenicity data in laboratory animals, which might be partly a consequence of acrolein’s toxicity, a possible role of acrolein in cigarette smoke-induced lung cancer has emerged from studies of its interactions with the *p53* tumor suppressor gene which mirror those found in lung tumors from smokers [27]. Acrolein is known to form exocyclic 1,*N* ^*2*^-deoxyguanosine adducts in DNA [23]. The mutagenicity of these adducts varies from none to moderate [24]. Multiple studies have detected acrolein-DNA adducts in human tissues or cells, including oral cells, colon cells, leukocytes, bladder mucosa, and lung, but there is presently no evidence that these adduct levels are higher in the lungs of smokers than non-smokers [26,61–64]. Collectively, these studies suggest a possible genotoxic role for acrolein in lung cancer induced by cigarette smoke, but this hypothesis has gaps. Acrolein could also contribute to lung cancer etiology by increasing inflammation in the lung [55], thus acting as a co-carcinogen by enhancing the consequences of DNA damage by carcinogens in tobacco smoke.

Crotonaldehyde, like acrolein, is a potent irritant to the respiratory tract and eyes. It caused preneoplastic lesions and a low incidence of neoplastic nodules and hepatocellular carcinoma in rats when administered in the drinking water [65]. Crotonaldehyde forms mutagenic cyclic 1,*N* ^*2*^-deoxyguanosine adducts in DNA [23,24,66], similar to those produced from acrolein, and these have been detected in human lung as well as other tissues [22,25,53,54].

Presumably, inhalation would be the relevant route of exposure for most of the effects discussed here, although it is possible that endogenous processes in the lung associated with the toxic effects of smoking could result in local generation of acrolein or crotonaldehyde. For example, oxidation of ω -3 fatty acids produces acrolein [67,68]; it is possible that oxidants in cigarette smoke interact with ω -3 fatty acids in the lung resulting in local formation of acrolein. There could also be unrecognized dietary or endogenous sources of acrolein and crotonaldehyde.

We observed higher levels of 3-HPMA and HMPMA in the urine of male than in female smokers, consistent with previously reported results of some [47], but not all studies of 3-HPMA [48]. Higher levels of a number of biomarkers including total nicotine equivalents in the urine of male smokers compared to female smokers have been observed in multiple previous studies, reflecting differences in smoking topography [47,69–71]. However, in our study, levels of 3-HPMA and HMPMA were higher in males than in females even after correcting for total nicotine equivalents. Consistent with the discussion above, this suggests that there is another source of acrolein and crotonaldehyde exposure which is greater in males than in females.

The relatively low levels of 3-HPMA and HMPMA in Latinos is also worth noting, as it is consistent with their relatively low lung cancer risk, in contrast to our previous observations of total nicotine equivalents and total NNAL in this group which were not significantly different from those of Whites. However, we also note that the Japanese-Americans levels of 3-HPMA in Model 2 (**Table 4, Fig 2**) are statistically indistinguishable from those of Whites, and this does not correlate with their lung cancer risk.

We did not observe a significant signal in the GWAS in search of common genetic variants associated with urinary levels of 3-HPMA in our population. Acrolein is known to be an excellent substrate for GSTP1 with K_cat_/K_m_ values of 92–350 mM^-1.s-1^ while GSTM1-1 and GSTA1-1 have less catalytic activity [72–74]. The K_cat_/K_m_ values for catalysis of glutathione conjugation of crotonaldehyde by these enzymes are less than 1/10^th^ those of acrolein [73,74]. The *GSTP1* gene, located on chromosome 11q13, is a polymorphic gene encoding variant proteins with moderate activities for catalysis of acrolein conjugation [74]. *GSTP*-null mice suffered increased bladder damage upon treatment with cyclophosphamide, which releases acrolein as a metabolite, although the overall excretion of 3-HPMA in these mice was unchanged [75]. Based on these data collectively, we might have expected to see a signal on chromosome 11, particularly in association with 3-HPMA, but this was not observed. We did see significant variants associated with HMPMA, with the strongest association on chromosome 12 (rs 55922880), located near the gene *TBX3*, but the relationship of this gene to crotonaldehyde metabolism is not clear at present. Overall, the results suggest that the formation of the glutathione conjugates of acrolein and crotonaldehyde that are metabolized to urinary 3-HPMA and HMPMA is mainly a non-enzyme catalyzed process. The uncatalyzed reactions of acrolein and crotonaldehyde with glutathione and related sulfhydryl compounds are well established [52,72].

In summary, the results of this study provide some intriguing new leads with respect to the possible role of the α,β-unsaturated toxicants acrolein and crotonaldehyde in lung cancer etiology in smokers. The relatively high levels of the acrolein and crotonaldehyde biomarkers 3-HPMA and HMPMA in the urine of Native Hawaiians, compared to the other groups in the MEC, is particularly interesting. Further studies are required to investigate endogenous or exogenous exposures to acrolein and crotonaldehyde that might account for these results.

## Supporting Information

S1 Table Median and interquartile range for measures of 3-HPMA and HMPMA, stratified by sex and race/ethnicity. (DOCX)

S2 Table Top 100 Associations for 3-HPMA in Overall analysis. (CSV)

S3 Table Top 100 Associations for HMPMA in Overall analysis. (CSV)

S4 Table Top 100 Associations for 3-HPMA in African Americans. (CSV)

S5 Table Top 100 Associations for 3-HPMA among Whites. (CSV)

S6 Table Top 100 Associations for 3-HPMA in Native Hawaiians. (CSV)

S7 Table Top 100 Associations for 3-HPMA in Latinos. (CSV)

S8 Table Top 100 Associations for 3-HPMA in Japanese Americans. (CSV)

S9 Table Top 100 Associations for HMPMA in African Americans. (CSV)

S10 Table Top 100 Associations for HMPMA in Whites. (CSV)

S11 Table Top 100 Associations for HMPMA in Native Hawaiians. (CSV)

S12 Table Top 100 Associations for HMPMA in Latinos. (CSV)

S13 Table Top 100 Associations for HMPMA in Japanese Americans. (CSV)

S1 Fig Quantile-Quantile plot of the GWAS results for 3-HPMA. Genome-wide significance is defined as the Bonferroni corrected 5% significance threshold (p-value< 5.0×10^−8^).
(JPG)

S2 Fig Quantile-Quantile plot of the GWAS results for HMPMA. Genome-wide significance is defined as the Bonferroni corrected 5% significance threshold (p-value< 5.0×10^−8^).
(JPEG)
