# Supplementary material for: Mercapturic Acids Derived from the Toxicants Acrolein and Crotonaldehyde in the Urine of Cigarette Smokers from Five Ethnic Groups with Differing Risks for Lung Cancer
Source: PLoS One. 2015 Jun 8;10(6):e0124841. doi: 10.1371/journal.pone.0124841 (PMC4460074; doi:10.1371/journal.pone.0124841)
Supplement: S1 Table — (DOCX) [file pone.0124841.s001.docx]

**Supplementary Table 1.** Median and interquartile range for measures of 3-HPMA and HMPMA, stratified by sex and race/ethnicity.

|  | **Males** | | | | **Females** | | | |  |
| --- | --- | --- | --- | --- | --- | --- | --- | --- | --- |
|  | **N** | **Median** | **(Interquartile  range)** | **p-value when compared to whites** | **N** | **Median** | **(Interquartile  range)** | **p-value when compared to whites** | **p-value comparing sex** |
| **3-HPMA (pmol/ml)** |  |  |  |  |  |  |  |  |  |
| **African Americans** | 111 | 5480 | (2652-9275) | 0.37 | 251 | 2895 | (1529.3-5993.4) | 0.73 | <0.0001 |
| **Native Hawaiians** | 120 | 5389 | (2803-8150) | 0.46 | 209 | 3002 | (1876.4- 5339.2) | 0.65 | <0.0001 |
| **Whites** | 190 | 4710 | (2388-8065) |  | 248 | 2983 | (1571.8-5275.6) |  | <0.0001 |
| **Latinos** | 235 | 3235 | (1426-5635) | <0.0001 | 214 | 2065 | (1077.4- 4226.8) | 0.0005 | 0.0002 |
| **Japanese Americans** | 402 | 3747 | (2062-6535) | 0.003 | 302 | 2321 | (1206.5- 4027.7) | 0.001 | <0.0001 |
| **p-value** |  |  | <0.0001 |  |  |  | <0.0001 |  |  |
|  |  |  |  |  |  |  |  |  |  |
| **HMPMA (pmol/ml)** |  |  |  |  |  |  |  |  |  |
| **African Americans** | 111 | 4303 | (2288-6327) | 0.15 | 250 | 2432 | (1231.3-4648.8) | 0.08 | <0.0001 |
| **Native Hawaiians** | 120 | 3738 | (1875-5512) | 0.81 | 209 | 2505 | (1363.4-3777.7) | 0.14 | 0.0002 |
| **Whites** | 190 | 3518 | (1787-5625) |  | 250 | 2111 | (1166.8-3600.5) |  | <0.0001 |
| **Latinos** | 236 | 2279 | (1286-3886) | <0.0001 | 216 | 1701 | (947.1-3332.7) | 0.0005 | 0.003 |
| **Japanese Americans** | 401 | 2399 | (1294-4102) | <0.0001 | 301 | 1541 | (861.2-2930.8) | 0.03 | <0.0001 |
| **p-value** |  |  | <0.0001 |  |  |  | <0.0001 |  |  |
|  |  |  |  |  |  |  |  |  |  |
|  |  |  |  |  |  |  |  |  |  |
| **3-HPMA/TNE (10^3^)** |  |  |  |  |  |  |  |  |  |
| **African Americans** | 111 | 97.65 | (61.13-172.0) | 0.05 | 251 | 77.74 | (47.54-115.7) | <0.0001 | 0.005 |
| **Native Hawaiians** | 120 | 129.5 | (105.1-200.1) | <0.0001 | 209 | 103.6 | (75.10-134.4) | 0.29 | <0.0001 |
| **Whites** | 190 | 108.1 | (79.96-147.4) |  | 248 | 93.75 | (63.31-140.7) |  | 0.01 |
| **Latinos** | 235 | 89.84 | (60.98-140.6) | 0.002 | 214 | 80.28 | (47.96-108.4) | <0.0001 | 0.003 |
| **Japanese Americans** | 402 | 125.7 | (87.41-178.0) | 0.01 | 302 | 94.60 | (66.67-145.0) | 0.65 | <0.0001 |
| **p-value** |  |  | <0.0001 |  |  |  | <0.0001 |  |  |
|  |  |  |  |  |  |  |  |  |  |
| **HMPMA/TNE (10^3^)** |  |  |  |  |  |  |  |  |  |
| **African Americans** | 111 | 74.46 | (47.26-113.5) | 0.08 | 250 | 58.69 | (38.65-85.01) | 0.0007 | 0.004 |
| **Native Hawaiians** | 120 | 94.29 | (71.31-130.8) | 0.01 | 209 | 76.33 | (59.92-104.7) | 0.05 | <0.0001 |
| **Whites** | 190 | 83.11 | (59.32-112.6) |  | 250 | 69.41 | (49.50-99.53) |  | 0.002 |
| **Latinos** | 236 | 65.90 | (45.14-102.3) | 0.05 | 216 | 62.67 | (40.99-93.89) | 0.03 | 0.14 |
| **Japanese Americans** | 401 | 84.28 | (59.76-116.5) | 0.91 | 301 | 70.98 | (50.42-99.34) | 0.99 | <0.0001 |
| **p-value** |  |  | <0.0001 |  |  |  | <0.0001 |  |  |
|  |  |  |  |  |  |  |  |  |  |
|  |  |  |  |  |  |  |  |  |  |
| **3-HPMA (pmol/ mg creatinine)** |  |  |  |  |  |  |  |  |  |
| **African Americans** | 111 | 4587 | (2706-6926) | <0.0001 | 251 | 3930 | (2215-6768) | <0.0001 | 0.23 |
| **Native Hawaiians** | 120 | 6667 | (4186-10,607) | 0.77 | 209 | 5732 | (3835-9217) | 0.28 | 0.08 |
| **Whites** | 190 | 6872 | (4354-10,540) |  | 248 | 6593 | (3600-10,608) |  | 0.25 |
| **Latinos** | 235 | 3705 | (1868-5908) | <0.0001 | 214 | 3320 | (1845-5940) | <0.0001 | 0.84 |
| **Japanese Americans** | 402 | 5536 | (3114-8732) | <0.0001 | 302 | 5048 | (3199-7998) | 0.002 | 0.36 |
| **p-value** |  |  | <0.0001 |  |  |  | <0.0001 |  |  |
|  |  |  |  |  |  |  |  |  |  |
| **HMPMA (pmol/mg creatinine)** |  |  |  |  |  |  |  |  |  |
| **African Americans** | 111 | 3357 | (2181-5160) | <0.0001 | 250 | 3153 | (17.098-54.312) | <0.0001 | 0.32 |
| **Native Hawaiians** | 120 | 4303 | (2530-7498) | 0.04 | 209 | 4456 | (27.998-66.382) | 0.65 | 0.66 |
| **Whites** | 190 | 4957 | (3361-7694) |  | 250 | 4606 | (29.624-70.47) |  | 0.13 |
| **Latinos** | 236 | 2487 | (1467-4519) | <0.0001 | 216 | 2771 | (15.784-46.086) | <0.0001 | 0.52 |
| **Japanese Americans** | 401 | 3546 | (2198-5427) | <0.0001 | 301 | 3604 | (21.22-55.38) | <0.0001 | 0.7 |
| **p-value** |  |  | <0.0001 |  |  |  | <0.0001 |  |  |

^a^ Includes never married, separated, widowed, and divorced.
