# Supplementary figures and images for: Mercapturic Acids Derived from the Toxicants Acrolein and Crotonaldehyde in the Urine of Cigarette Smokers from Five Ethnic Groups with Differing Risks for Lung Cancer
Source: PLoS One. 2015 Jun 8;10(6):e0124841. doi: 10.1371/journal.pone.0124841 (PMC4460074; doi:10.1371/journal.pone.0124841)

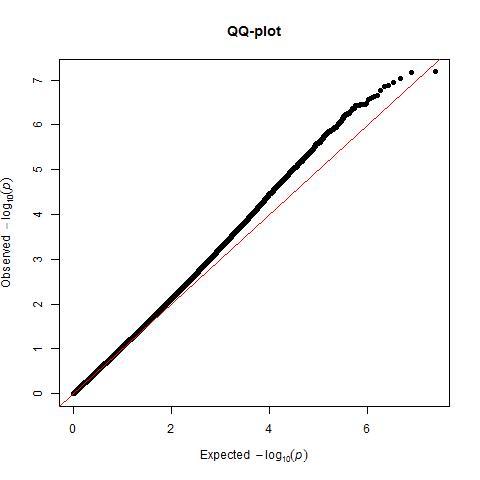

Supplement: S1 Fig — Genome-wide significance is defined as the Bonferroni corrected 5% significance threshold (p-value< 5.0×10^−8^). (JPG) [file pone.0124841.s014.jpg]

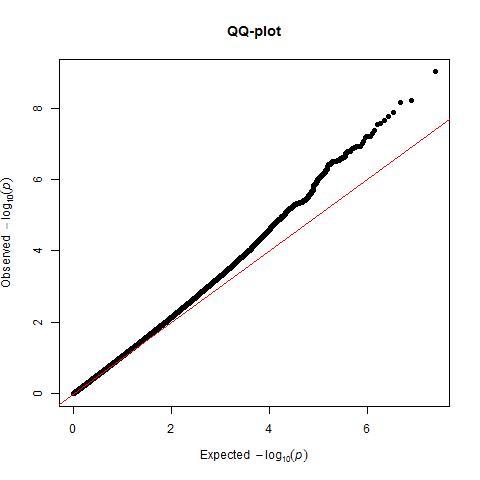

Supplement: S2 Fig — Genome-wide significance is defined as the Bonferroni corrected 5% significance threshold (p-value< 5.0×10^−8^). (JPEG) [file pone.0124841.s015.jpeg]
